# The blood microbiome and its association to cardiovascular disease mortality: case-cohort study

**DOI:** 10.1186/s12872-022-02791-7

**Published:** 2022-07-31

**Authors:** Graeme Lawrence, Ingvild Midtervoll, Sven Ove Samuelsen, Anne Karin Kristoffersen, Morten Enersen, Lise Lund Håheim

**Affiliations:** 1 Department of Mathematics, Faculty of Mathematics and Natural Sciences, University of Oslo, Oslo, Norway; 2 Institute of Oral Biology, Faculty of Dentistry, University of Oslo, Blindern, P.O.B. 1052, 0316 Oslo, Norway

**Keywords:** Blood bacteria, *16SrRNA*, Mortality, Cardiovascular disease, Case-cohort study, Proportional hazards regression

## Abstract

**Background:**

Little is known about the association between bacterial DNA in human blood and the risk of cardiovascular disease (CVD) mortality.

**Methods:**

A case-cohort study was performed based on a 9 ½ year follow-up of the Oslo II study from 2000. Eligible for this analysis were men born in 1923 and from 1926 to 1932. The cases were men (n = 227) who had died from CVD, and the controls were randomly selected participants from the same cohort (n = 178). Analysis of the bacterial microbiome was performed on stored frozen blood samples for both cases and controls. Association analyses for CVD mortality were performed by Cox proportional hazard regression adapted to the case-cohort design. We used the Bonferroni correction due to the many bacterial genera that were identified.

**Results:**

Bacterial DNA was identified in 372 (82%) of the blood samples and included 78 bacterial genera from six phyla. Three genera were significantly associated with CVD mortality. The genera *Kocuria* (adjusted hazard ratio (HR) 8.50, 95% confidence interval (CI) (4.05, 17.84)) and *Enhydrobacter* (HR 3.30 (2.01, 5.57)) indicate an association with CVD mortality with increasing levels. The genera *Paracoccus* (HR 0.29 (0.15, 0.57)) was inversely related. Significant predictors of CVD mortality were: the feeling of bad health; and the consumption of more than three cups of coffee per day. The following registered factors were borderline significant, namely: a history of heart failure; increased systolic blood pressure; and currently taking antihypertensive drugs now, versus previously.

**Conclusions:**

The increasing levels of two bacterial genera *Kocuria* (skin and oral) and *Enhydrobacter* (skin) and low levels of *Paracoccus* (soil) were associated with CVD mortality independent of known risk factors for CVD.

**Supplementary Information:**

The online version contains supplementary material available at 10.1186/s12872-022-02791-7.

## Background

Several studies have documented an association between chronic infections and the risk of cardiovascular diseases (CVD) [1–3]. The circulation is a closed system, and the blood in healthy individuals was earlier believed to represent a sterile environment, which is the basis for safe blood transfusions [4]. However, Nikkari et al. reported already the presence of bacterial DNA in healthy human blood in 2001, and Moriyama et al. defined a healthy human blood microbiome in 2008 [5, 6]. In an overview of blood microbiome studies, Castilla and coworkers described how the blood microbiome may interact with other human microbiomes [7].

All of the biological processes by which bacteria may influence the circulation are not clarified, but the main processes are atherosclerosis and thrombosis. Atherosclerosis is well known as a low-grade, chronic inflammation of the arterial wall, and is an important factor in the development of several diagnoses of CVD. For many years bacteria have been suspected to be part of the pathogenesis of this group of diseases, as whole bacteria, fragments and their DNA have been identified in blood from CVD patients [2, 3, 8–10]. Several other key processes may be responsible for atherosclerosis; among them accumulation of monocyte/macrophage lineage cells within the lipid-rich subendothelial space of the affected artery where bacterial lipopolysaccharide (LPS) also participate in the formation of macrophage-derived foam cells [11, 12]. Accumulation of lipid bodies in the foam cells is affected by both the nutrition and gut microbiota. Bacteria are also known to form thrombi and emboli in interaction with platelets, and this is also seen as a complication of advanced lesions of atherosclerosis [1–3].

Gut bacteria may act as key “metabolic filters” of the diet. They can convert common nutrients to metabolites, as specific microbial-associated metabolites, such as trimethylamine-N-oxide (TMAO) and short-chain fatty acids (SCFAs). These components including secondary bile acid have also been shown to affect the progression of CVD. Furthermore, gastrointestinal infection or autoimmune diseases such as gluten intolerance may contribute to the leakage of bacteria into the circulation and promote atherosclerosis [13, 14]. Bacterial DNA identified in blood or serum may represent live bacteria, cultivable or uncultivable bacterial species as well as dormant (non-dividing) bacteria [15]. Live bacteria and their membrane vesicles may enter the bloodstream via leaking epithelial junctions or mucosal disruptions [16]. The lung microbiome has also been defined and shown how it may change during disease [17]. The constitution of the respiratory microbiome is determined by three factors: microbial immigration, elimination, and the relative reproduction rates of its members. Those factors may also influence the blood microbiome during CVD.

In patients with untreated advanced periodontal disease, chewing and tooth brushing may also result in chronic bacteraemia by migration of bacteria from the subgingival biofilm through the junctional epithelium and into blood vessels in the connective tissue [18]). In a study by Håheim et al., it was indicated that a reduced level of antibodies to oral bacteria in chronic periodontitis may permit the spread of bacteria in the circulation and increase the risk of cardiovascular disease mortality [19]. In a randomized controlled trial, non-surgical mechanical periodontal therapy significantly reduced systemic levels of C-reactive protein, fibrinogen and white blood cells in cases as compared to controls after 2 months of follow-up [20].

Risk factors most commonly associated with CVD include modifiable risks—such as tobacco use, obesity, diet, and physical inactivity. However, little is known about whether certain bacteria in the blood microbiome are correlated with these known risk factors for CVD [3]. More scientific knowledge on the role of the bacterial microbiome can improve public health. Low-grade inflammation is known to be part of atherosclerosis and knowledge of the role of the blood microbiome may potentially lead to primary and/or secondary preventive measures to decrease the incidence and mortality of CVD. The aim of this study was to study whether identified bacterial DNA (*16S rRNA*) in blood samples from elderly men was associated with cardiovascular disease mortality in relation to known risk factors in a 9 ½ year follow-up using a case-cohort study design.

## Methods

### Study cohort

This study is based on a selection of cases and controls of 405 individuals that participated in the Oslo II study in 2000, constituting a case-cohort study within a 9 ½ years follow-up study [21]. The Oslo II study was the second screening of the Oslo study of 1972/73. This was a study of men and the risk factors of CVD with long-term follow-up on CVD and mortality. The cases are men that died from cardiovascular diseases in the period from 2000, and control persons are a random selection of men alive by the end of follow-up, 31^st^ December 2009. In short, 12,764 men were invited to the Oslo II study in 2000 that had either previously taken part in or had been invited but not taken part in the Oslo study of 1972/73 (n = 17,965 participants). Among the participants of the Oslo II study 5323 men attended both health screenings and 1207 attended in 2000 only, totalling 6530 participating men in 2000, born in the period 1923–1952. Of these 5503 were born in the years 1923 or 1926–1932. All participants gave their written consent to the use of the data and biological material on the condition that The Data Inspectorate and The Regional Ethical Review Board for Medical Research, Eastern Norway, had granted the permission. The Oslo II-study followed the ethical principles of the Helsinki Declaration for Medical Research involving persons.

### Data collection

Health information of the participants was provided by questionnaire information, anthropologic measurements, and full blood and serum sample analyses. Serum samples and EDTA full blood were stored at -80^O^C for future research projects.

### Definition of outcome

Causes of mortality were selected by the ICD10 classification as provided by Statistics Norway. The selected diagnoses were myocardial infarction, stroke, aneurysm and if needing more cases, ischaemic heart disease. The data linkage made use of the personal identifier number which all Norwegians have. Statistics Norway provided the data on the cause of death from the time of screening in 2000 to the end of the follow-up period, December 31st 2009, through one direct data linkage.

### Case-cohort study group

The population of this case-cohort sub-study was selected among men born in the years 1923 or 1926 to 1932 (n = 5503) (Table 1). The cases of this case-cohort study were selected on grounds of mortality from cardiovascular disease (n = 227). The control persons (n = 178) were randomly selected from the cohort of 5503–227 = 5276 non-case men of these age groups who had participated in the health screening in 2000. The controls were sampled after the end of follow-up in 2010. Thus we were able to sample a control group not overlapping the cases. The study is thus a variation of the case-cohort design as discussed by Chen in 2001 and Samuelsen et al. in 2007 [22, 23]. The analyses were carried out in accordance with those of a standard case-cohort design.

**Table 1:** Birth years for cases and controls of the case-cohort study and the full cohort they were drawn from

Birth year	Controls	Cases	Full cohort
1923	21	3	572
1924	0	0	0
1925	0	0	0
1926	21	56	675
1927	22	34	640
1928	18	36	710
1929	25	34	698
1930	24	29	746
1931	26	18	722
1932	21	17	740
Total	178	227	5503

### Covariates

At the screening, total serum cholesterol, HDL-cholesterol, glucose non-fasting and triglycerides were measured in serum. Body Mass Index (BMI) was calculated as Kg/m^2^. Systolic and diastolic blood pressures were measured (mean value of the two last of three recordings). Alcohol drinking patterns and other covariates mentioned below were obtained from the questionnaire. The response alternatives for alcohol consumption per week were: 4–7 times, 2–3 times, or about once; otherwise less frequent alternatives: 2–3 times or about once per month, a few times during the last year, drank alcohol last year, or never drink alcohol. Further, daily smoking was recorded as yes/no. Diabetes was recorded as Type 1, Type 2 or non-diabetic. The level of education, in years, was used as a proxy for socioeconomic status in the analyses. Also measured were: subjective health, excessive coffee consumption (more than 3 cups per day), and level of physical activity at leisure. These factors were included as covariates in the explorative statistical analyses. Several of these factors are known to be potential confounders to different diagnoses due to atherosclerosis and/or thrombosis in CVD [3, 21].

### Sample analyses, collection, DNA isolation, PCR amplification and gene sequencing

The blood samples from the screening in 2000 had been stored at the HUNT biobank in Levanger, Norway (https://www.ntnu.edu/hunt/hunt-biobank). At the HUNT biobank, the selected blood samples for the case-cohort study were prepared for the DNA isolation. Each sample was thawed in a water bath at 20 °C for 40 min before 700 µl blood was transferred to a tube containing 700 µl RNA (Ambion cat.no AM7020), and stored at -20 °C until the time of DNA isolation. The samples were transported to the Institute of Oral Biology, Faculty of Dentistry, University of Oslo, under optimal storing conditions on dry ice. Handling and analyses of all samples were performed blinded to patient diagnosis and outcome.

DNA from the samples was extracted using a MagnatrixOS 1200 robot and a Qiagen MagAttract DNA blood M48 kit (Qiagen GmbH, Hilden, Germany), according to the manufacturer’s protocol. Sterile molecular grade water was used for negative controls. *16S rRNA* gene amplification was performed using primers flanking the V3 and V5 regions: E334F 5′-CCAGACTCCTACGGGAGGCAGC-3′ and E939R 5′-CTTGTGCGGGCCCCCGTCAATTC-3′. The PCR, library preparation and sequencing were performed in 2014–2015 in parallel [24]. Sequencing data are available through NCBI’s sequencing read archive (SRA) with the accession numbers SRP134262 and SRP134259 for the samples investigated, and SRP134257 for the negative controls. A supporting file containing information on library preparation and sequencing procedure in the Roche Junior Sequencer is included as supplemental material (See Additional file 1).

### Statistical analyses

The difference between cases and controls for the included risk factors was tested using two-sided t-tests for quantitative measures and Fisher exact tests for proportions. Cox-regression for proportional hazards models, adapted to the case-cohort design by inverse probability weighting, was performed allowing the computation of hazard ratios and corresponding 95% confidence intervals, the latter of which using robust variances [25–27]. Covariates for all cases were obtained and cases were thus given weights of 1, while controls were assigned larger weights, 5276/178 = 29.64, equal to the inverse of the inclusion probability of a non-case being sampled. Individuals' ages were used as survival times rather than observed time from entry in order to negate age as a confounding risk factor. The data were thus left truncated with entry time age in the year 2000 and exit time age at death or emigration if this occurred before the year 2010 and age in 2010 if not. The event indicator was equal to the cases indicator. Cumulative incidences of CVD, i.e. probabilities of dying from the specified causes, were calculated in accordance with the case-cohort design. To be included in the analyses a genus needed to be present in at least five samples of cases and controls, as well as over five per cent of the total study population. The 5% decision on the patient population was made to avoid unstable and possibly infinite estimates for estimated regression parameters (= log(hazard ratios)). We were also concerned that the large sample properties used for confidence intervals of hazard ratios would not be valid in such a small data set. In particular, to be able to carry out the elaborate model selection schemes, we chose this restriction to avoid numerical instability during the selection steps. Computation was done with the statistical package R, using weights in the *coxph* function of the *survival* package [28].

Preliminary univariate analyses of the data were undertaken by including only one variable in the adapted Cox model at a time. Those variables that obtained a *p*-value below 0.05, were considered statistically significant to be included in the multivariate analyses. Several multivariate models were then created, via stepwise selections based on various stopping criteria and a Lasso shrinkage method, in order to adjust the bacteria HRs for known risk factors. The final model was arrived at by minimizing an AIC measure modified for weighted case-cohort analysis. The other approaches generally led to similar models.

A Bonferroni correction of α = 0.05/78 = 0.0006 was applied, as the hypotheses of 78 genera were tested. The work is part of a Master’s Thesis in Statistics at the University of Oslo [29].

## Results

The 227 cases died from different cardiovascular diseases; 113 from ischaemic heart disease (ICD-10 codes I219-I259) of which 84 were from acute or subsequent myocardial infarction and 39 from chronic ischaemic heart disease, 83 from cerebrovascular disease (ICD-10 codes I609-I64), 21 from aortic aneurysm and dissection (ICD-10 codes I710-I719). The age distribution is shown in Table 1.

There were distinct differences in baseline characteristics among cases and controls (Table 2). It was noticeable that among the cases more men had diabetes than among control persons (17.3% vs 6.8%). Over half the cases (53.2%) took antihypertensive medication, while only a quarter (25.0%) of controls did. Among the cases, 29.7% reported having experienced myocardial infarction and 9.1% heart failure, versus 10.1% and 4.7% respectively among controls. More cases also responded to having bad health (5.8% vs 2.8%) and 56.2% of cases reported to not taking part in hard physical exercise compared to 36.8% of control persons. A significant difference was found in the number of diabetes sufferers (*p*-value 0.002), antihypertensive medication users (< 0.001), those with a history of myocardial infarction (< 0.001) and men who engaged in no hard physical activity (0.002).

**Table 2:** Baseline characteristics of study participants; cases, controls, and combined sample

Variable	Cases n = 227	Controls n = 178	Combined sample n = 405	t-test/Fisher test *p*-value (2-sided)
Age at entry, mean (SD) years	71.3 (2.0)	71.2 (2.7)	71.3 (2.4)	0.755
Age at exit, mean (SD) years	76.9 (3.5)	80.2 (3.2)	78.4 (3.7)	0.568
Diabetes, yes	38 (17.3%)	12 (6.8%)	50 (12.6%)	0.002
Use antihypertensive medication, yes	117 (53.2%)	42 (25.0%)	159 (41.0%)	< 0.001
History of myocardial infarction, yes	66 (29.7%)	18 (10.1%)	84 (21.0%)	< 0.001
History of heart failure, yes	20 (9.1%)	8 (4.7%)	28 (7.2%)	0.114
Systolic blood pressure, mean (SD) mmHg	151.0 (22.2)	145.0 (20.5)	148.4 (21.7)	0.253
Subjective health, bad	13 (5.8%)	5 (2.8%)	18 (4.5%)	0.224
Physical activity, no hard activity	125 (58.7%)	70 (43.5%)	195 (52.1%)	0.002
Excessive coffee consumption, > 3 cups per day	91 (40.4%)	54 (31.2%)	145 (36.4%)	0.047

Nine years follow-up of the Oslo II study from 2000

The microbiological analyses yielded a multitude of different bacterial genera present to a varying degree in the blood. In total 484 different bacteria sequences were identified in 372 blood samples, belonged to 88 different genera. Ten genera were found in very few samples, and therefore 78 genera were included in univariate analyses. These were included in univariate analyses, and those that were found to be significant were then considered for multivariate analyses. Table 3 presents those genera found to be associated with CVD mortality in the univariate analyses. The most prevalent genus among cases was *Streptococcus* with 42.3% prevalence (49.5% in total sub-cohort), the second was *Staphylococcus* with 38.3% versus 32.6%, and the third most common was *Enhydrobacter* with 34.8% versus 25.4%, respectively. Other noticeable genera were *Kocuria, Paracoccus, Stenotrophomonas, Veillonella,* and *Bacteroides.* Periodontal related genera as *Porphyromonas, Tannerella* and *Prevotella* were found in both case and control groups but the respective prevalence was lower than that of Bacteroides.

**Table 3:** Prevalence of bacterial genera amongst cases and controls sorted according to total prevalence in the subsample

Genus	Case prevalence (%)	Control prevalence (%)	HR	Confidence interval 95%	*p*-value
*Streptococcus*	42.3	58.9	0.54	0.36–0.81	0.003
*Staphylococcus*	38.3	25.1	1.70	1.09–2.57	0.020
Enhydrobacter*	34.8	13.1	3.30	2.01–5.57	< 0.001
Kocuria*	22.5	3.4	8.50	4.05–17.84	< 0.001
Paracoccus*	5.7	18.3	0.29	0.15–0.57	< 0.001
*Stenotrophomonas*	7.5	13.7	0.48	0.25–0.92	0.028
*Veillonella*	4.9	12.0	0.32	0.15–0.68	0.003
*Bacteroides*	2.6	10.9	0.24	0.09–0.63	0.004

Hazard ratios for cardiovascular disease mortality by Cox proportional hazard regression univariate analyses are presented with accompanying confidence intervals and *p*-values for the bacteria found to be significant at the 2% cut-off point. Each genus must be present in at least five cases and five controls as well as over five per cent total prevalence to be included in the analyses

*The bacterial genus is also significant when considering the Bonferroni correction

Eight genera were further explored in follow-up analyses (Table 4). Increasing levels of bacterial DNA for *Staphylococcus, Enhydrobacter,* and *Kocuria* were associated with CVD mortality whilst there was an inverse relationship for *Streptococcus, Paracoccus, Stenotrophomonas, Veillonella,* and *Bacteroides.* The latter four genera *Paracoccus, Stenotrophomonas, Veillonella,* and *Bacteroides* had lower levels among cases versus the total. In the final multivariate model, the two genera *Enhydrobacter* and *Kocuria* were positively associated while *Stenotrophomonas, Bacteroides* and *Paracoccus* were negatively related to CVD mortality. *Streptococcus* was non-significant and *Veillonella* was also no longer in the full model*.* Only *Enhydrobacter, Kocuria,* and *Paracoccus* were significant when considering the Bonferroni correction.

**Table 4:** Hazard ratios with corresponding 95% confidence intervals and *p*-values for the variables selected in the final model of the Cox proportional hazards regression multivariate analyses for cardiovascular disease mortality

Variable	Hazard ratio	Confidence interval 95%	*p*-value
*Bacterial genus*			
*Kocuria*	3.60	1.62–7.78	0.002
*Enhydrobacter*	3.20	1.68–5.91	< 0.001
*Bacteroides*	0.22	0.07–0.71	0.011
*Paracoccus*	0.34	0.15–0.77	0.010
*Stenotrophomonas*	0.35	0.16–0.77	0.009
*Streptococcus*	0.76	0.46–1.25	0.276
*General health*			
Systolic blood pressure, 10 mmHg	1.10	0.99–1.24	0.066
Diabetes, yes	3.10	1.51–6.22	0.002
History of heart failure, yes	2.30	0.88–5.81	0.089
History of myocardial infarction, yes	2.20	1.25–4.01	0.007
Hypertensive medication, yes versus never	2.20	1.27–3.65	0.004
Hypertensive medication, yes versus previous	3.20	0.82–12.79	0.093
*Lifestyle factor*			
Subjective health, not so good versus bad	0.20	0.08–0.50	0.001
Subjective health, good versus bad	0.13	0.05–0.32	< 0.001
Subjective health, very good versus bad	0.06	0.02–0.19	< 0.001
Excessive coffee consumption, > 3 cups per day	1.70	1.06–2.75	0.029

An investigation into the correlation between bacterial genera revealed that two, *Kocuria* and *Enhydrobacter,* were significantly correlated with a Pearson’s correlation coefficient of 0.550. This relationship was further explored by creating a multivariate model that considered the interaction of the two genera; which was found not significant (*p* = 0.054). The assumption that only the individual effects of *Kocuria* and *Enhydrobacter* are significant is therefore maintained.

In addition to bacterial genera, general health parameters and lifestyle factors were analysed (Table 4). The covariates in the final statistical model are those reported in Table 4. Subjective health was inversely related for all three levels of comparison: not so good, good and very good, versus bad. Coffee consumption of more than three cups of coffee per day increased the risk of CVD mortality in this case-cohort adapted Cox multivariate analysis. History of heart failure, systolic blood pressure, and currently taking antihypertensive were borderline significant.

**Fig. 1:**
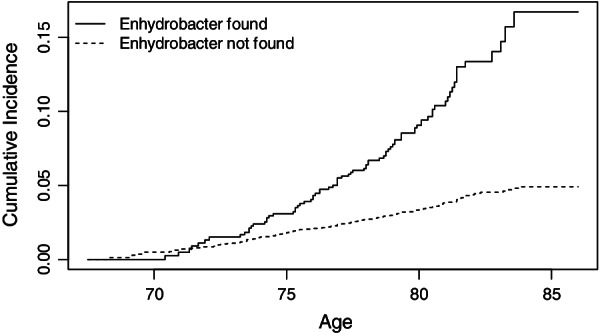
Cumulative incidence of CVD from age 67 among subjects with and without *Enhydrobacter* detected

In Fig. 1 we show the cumulative incidence of CVD comparing individuals where *Enhydrobacter* was and was not detected. The figure displays the probability of dying from the causes from the lowest entry time of 67 years up to the highest exit time of 87 years. In accordance with the HR 3.30, we see that the probabilities are higher for individuals with *Enhydrobacter*. Similar figures for this genera and the other seven genera are included in the supplement (see Additional file 2).

## Discussion

Three bacterial genera were found to be associated with mortality of the selected diagnoses of CVD adjusted for factors of general health and lifestyle in the final multivariate model. *Enhydrobacter* (HR 3.30, 95% CI 2.01–5.57) and *Kocuria* (HR 8.50, 4.05–17.84) were associated with increased risk and *Paracoccus* (HR 0.29, 0.15–0.57) with decreasing risk. These were the genera that were also significant after Bonferroni correction. The most prevalent genera *Staphylococcus* (HR 1.70, 1.09–2.57) and *Streptococcus* (HR 0.54, 0.36–0.81) were not found to be significantly associated in the multivariate analysis nor were *Veillonella* (HR 0.32, 0.15–0.68).

*Enhydrobacter* is a member of the phylum *Proteobacteria* (alpha) with only one known species, *aerosaccus*. It is a gram-negative rod, non-motile, facultative aerobic and most common on the skin [30]. *Kocuria* belongs to the phylum *Actinobacteria* and is a gram-positive cocci, facultative anaerobe and lives on the skin and in the oral cavity generally. It is considered to be non-pathogenic but occurs in some oropharyngeal infections, as well as in endocarditis [31]. *Paracoccus* belongs to the phylum Proteobacteria and is a gram-negative non-motile cocci. It is found in soil in both aerobic and anaerobic environments and is an opportunistic pathogen [32].

The *Staphylococcus* and *Streptococcus* genera were detected in the case and in the control groups without significant differences (Table 4). The reason why these genera did not predict mortality from CVD could be their role as part of the commensal microbiota living in homeostasis with the host. However, in some individuals, the immune system does not protect individuals, and serious disease as infective endocarditis and rheumatic heart disease may develop. Furthermore, microbiological investigations of tissue samples from acute cases of CVD have reported associations of the periodontal pathogens to CVD [33, 34].

The participants of this study were older men with mean age of 70 years at the start of the study, and they were followed up with regard to mortality for nine and a half years. Exposure to bacteria and mortality are expected to increase with age. The occurrence and consequences of infections may be more obvious at a later age. Only men were included which limits the generalizability of the results. Statistically, the main strength of the study was the results of three genera being significantly associated with CVD mortality after the Bonferroni adjustment. The bacterial identification was done without the knowledge of case status. Importantly, the outcome mortality data was provided by Statistics Norway and the National death registry administered by the Norwegian Institute for Public Health. We did not sequence the full genome in the relevant *16SrRNA* genome but the so-called V3-4 and V3-5 sections. The only option in the analysis here was to do the analysis at the genus level, to keep enough power. Furthermore, our detection at the species level of *Kocuria*, *Enhydrobacter* and *Paracoccus* here will only be descriptive and not part of any survival analyses.

The genera identified here are normal inhabitants of human niches as the skin, the gut, the oral cavity, and the soil. The bacteria may have entered the bloodstream via epithelial cell junctions (leaking), mucosal disruptions in the gut, the oral cavity, or through disruptions in the skin mucosa. Although each genus represents several species, these analyses indicate which genera were significantly associated with CVD. Some of the periodontal pathogens belong to the genera *Porphyromonas* and *Prevotella*, and there were not detected any associations between these genera and CVD in our analysis. Furthermore, our analysis on genus level for reasons explained earlier, did not indicate signs of the red complex in the blood samples [35, 36]. However, antibodies to these bacteria have been analysed in a separate study of Oslo II as reported previously [19]. Different aspects of oral infections and cardiovascular disease have also been explored broadly in an earlier textbook [37].

The *Enhydrobacter*, *Kocuria* and *Paracoccus* genera all comprise species reported to be associated with human opportunistic infections. Bacterial genera induce the immune system to a different degree. Xuanzhi et al. recently published a review giving an overview of pathological pathways for bacterial infections being involved in atherosclerosis [38], which include a number of virulence factors that trigger the immune system and cell death in the development of atherosclerosis. Bacterial DNA has been identified in the blood, in the atrial wall, and in heart valves [22]. An immunological reaction to bacteria is also the production of antibodies [21]. Oral bacteria have been associated with changes in inflammatory parameters [36, 37].

This study reflects the risk through a lifetime as it is a study of elderly men. The results have therefore been adjusted for history of CVD in order to find whether the results are independent of CVDs as myocardial infarction, ischaemic heart disease, heart failure, and stroke including the effect of other risk factors and can be seen as intermediate factors in a causal analysis. SBP and antihypertensive medication were independently associated with CVD mortality in this study. When comparing studies different factors may play a part: (1) The genera/species may not be the same in other populations, (2) Have the full microbiome been studied or selected genera? (3) We cannot distinguish between viable or dead bacteria in our study. Bacterial findings can be from infections handled by the immune defence. The value of this study is the hypothesis generating results of which genera to pursue in future research projects.

## Conclusions

This study shows that a range of bacterial genera can be identified in the blood with origin from the gut, the skin, the oral cavity and the environment. Three genera, *Enhydrobacter, Kocuria,* and *Paracoccus* (inversely*)* of skin and oral, skin, and soil microbiomes, respectively, were found to be associated with CVD mortality, and they were independent of other known covariates for CVD. The most prevalent genera *Staphylococcus* and *Streptococcus* were not significantly associated with CVD mortality using Bonferroni correction.

## Supplementary Information

**Additional file 1**. Library preparation and sequencing procedure in the Roche Junior Sequencer. The file contain information of library preparation and sequencing procedure in the Roche Junior Sequencer.

**Additional file 2**. Figures of the eight main bacterial genera and CVD. The file contain eight figures of the cumulative incidence of CVD comparing individuals where eight different bacterial genera were and were not detected. The figure displays the probability of dying from the causes from lowest entry time 67 years up to highest exit time 87 years.

## Data Availability

The Oslo II dataset includes participant information that is considered to be sensitive information. The data may be available on request, however, this is a composite data set with data from different registries and require permission from these and the Regional Committees for Medical and Health Research Ethics (REC South East). For information contact the host institution, the University of Oslo: eksp-iob@odont.uio.no.

## Abbreviations

CVD: Cardiovascular diseases
DNA: Deoxyribonucleic acid
CI: Confidence interval
RNA: Ribonucleic acid
LPS: Lipopolysaccharide
TMAO: Trimethylamine-N-oxide
SCFA: Short chain fatty acid
EDTA: Ethylenediaminetetraacetic acid
ICD: International codes of diseases
HDL: High-density lipoprotein
BMI: Body mass index
PCR: Polymerase chain reaction
HUNT: The Trøndelag Health study
SRA: Sequencing read archive
SRP: Sequencing read study
NCBI: National Center for Biotechnology Information
SD: Standard deviation
HR: Hazard ratio
