# Supplementary material for: The blood microbiome and its association to cardiovascular disease mortality: case-cohort study
Source: BMC Cardiovasc Disord. 2022 Jul 31;22:344. doi: 10.1186/s12872-022-02791-7 (PMC9339179; doi:10.1186/s12872-022-02791-7)
Supplement: Supplementary file 1 — Additional file 1. Library preparation and sequencing procedure in the Roche Junior Sequencer. The file contain information of library preparation and sequencing procedure in the Roche Junior Sequencer. [file 12872_2022_2791_MOESM1_ESM.docx]

**Library preparation and sequencing procedure in the Roche Junior Sequencer**

A second PCR with the index fusion adaptor-primer A (for Roche GS junior instrument) with *16S* *rRNA* 334F sequence and adaptor-primer B (for Roche GS junior instrument) with *16S* *rRNA* 939R sequence was performed with 20 cycles. The amplicons were purified by using Agencourt Ampure Beads (Agencourt Bioscience Corporation, Beckman Coulter company, Inc, Beverly, MA, USA) followed by DNA quantitation and quality examination using 2100 Bioanalyzer and the High Sensitivity DNA Assay kit (Agilent Technologies, Santa Clara, CA, USA). The final amplicon preparation products were used in emulsion PCR via Roche GS Lib-L kit (Roche Diagnostics Gmbh, Mannheim, Germany) with the use of molecules-per-bead ratio of 0.7. The emulsion PCR, library bead purification, and sequencing on 454 GS Junior Roche system were performed according to the manufacturer.

## Data processing and taxonomic classification

The analysis of the raw data from the Roche 454-sequencing followed a workflow based on the QIIME 1.8.0 (Quantitative Insights Into Microbial Ecology) pipeline (Caporaso *et al.*, 2010). Pyrosequencing data file (fasta) was demultiplexed with the command split_library.py with restriction in read length removal of reads smaller than 300 bases and larger than 600 bases. The command denoise_wrapper.py was used to qualify the correct signalling bases in the sequence. Chimera filtering was performed by UCHIME (Edgar *et al.*, 2011). Taxonomic analysis was performed using the “open_reference_otus.py” command with uclust alignment including de novo OTU picking and *16S rRNA* from Silva reference database (SSU Ref NR 119) and with *16S rRNA* RefSeq Version 13.2 Human oral microbiome database (HOMD).

All sample sequences were also analysed through the SILVAngs (Quast et al. 2013) to perform visualized overview of the taxa diversity in each group and to compare with the QIIME analysis.

1. Baker GC, Smith JJ, Cowan DA. Review and re-analysis of domain-specific 16S primers. J Microbiol Methods. 2003;55(3):541-55.
2. Wang Y, Qian PY. Conservative fragments in bacterial 16S rRNA genes and primer design for 16S ribosomal DNA amplicons in metagenomic studies. PLoS One. 2009;4(10):e7401.
3. Caporaso JG, Kuczynski J, Stombaugh J, Bittinger K, Bushman FD, Costello EK, et al. QIIME allows analysis of high-throughput community sequencing data. Nat Methods. 2010;7(5):335-6.
4. Robert C Edgar 1, Brian J Haas, Jose C Clemente, Christopher Quince, Rob Knight. UCHIME improves sensitivity and speed of chimera detection. Bioinformatics. 2011 Aug 15;27(16):2194-200.
5. Quast C, Pruesse E, Yilmaz P, Gerken J, Schweer T, Yarza P, Peplies J, Glöckner FO (2013) The SILVA ribosomal RNA gene database project: improved data processing and web-based tools. Opens external link in new window. Nucl Acids Res. 41 (D1): D590-D596.
