# Supplementary material for: The blood microbiome and its association to cardiovascular disease mortality: case-cohort study
Source: BMC Cardiovasc Disord. 2022 Jul 31;22:344. doi: 10.1186/s12872-022-02791-7 (PMC9339179; doi:10.1186/s12872-022-02791-7)
Supplement: Supplementary file 2 — Additional file 2. Figures of the eight main bacterial genera and CVD. The file contain eight figures of the cumulative incidence of CVD comparing individuals where eight different bacterial genera were and were not detected. The figure displays the probability of dying from the causes from lowest entry time 67 years up to highest exit time 87 years. [file 12872_2022_2791_MOESM2_ESM.docx]

**Additional file 2:**

**Figures of the eight main bacterial genera to CVD**

Figures depicting cumulative incidence of CVD with and without genera of bacteria detected.

**Figure 1. Cumulative incidence of CVD from age 67 among subjects with and without *Enhydrobacter* detected.**

**Figure 2. Cumulative incidence of CVD from age 67 among subjects with and without *Bacterioides* detected.**

**Figure 3. Cumulative incidence of CVD from age 67 among subjects with and without *Kocuria* detected.**

**Figure 4. Cumulative incidence of CVD from age 67 among subjects with and without *Paracoccus* detected.**

**Figure 5. Cumulative incidence of CVD from age 67 among subjects with and without *Staphylococcus* detected.**

**Figure 6. Cumulative incidence of CVD from age 67 among subjects with and without *Stenotrophomanas* detected.**

**Figure 7. Cumulative incidence of CVD from age 67 among subjects with and without *Streptococcus* detected.**

**Figure 8. Cumulative incidence of CVD from age 67 among subjects with and without *Veillonella* detected.**
